# Unveiling the Malignancy Risk of Surgically Treated Bethesda III Thyroid Nodules: A 10-Year Retrospective Study Comparing Fine-Needle Aspiration Cytology (FNAC) and Histopathology

**DOI:** 10.7759/cureus.75855

**Published:** 2024-12-17

**Authors:** Minhaj Rafi, Muhammad Awais Kanwal, Muhammad Awais, Taha Ahmad, Muhammad Tayyab, Muhammad Saulat Naem, Shayan K Ghaloo, Syed Raza Hussain, Muhammad Faisal

**Affiliations:** 1 Department of Surgical Oncology, Shaukat Khanum Memorial Cancer Hospital and Research Centre, Lahore, PAK

**Keywords:** atypia of undetermined significance (aus), atypia of undetermined significance (aus) or follicular lesion of undetermined significance (flus), aus/flus, bethesda iii, fnac, follicular lesion of undetermined significance (flus), histopathology, thyroid cancer, thyroid nodules

## Abstract

Introduction

Thyroid malignancy remains a significant global health concern, making the accurate differentiation between benign and malignant thyroid nodules crucial for optimal patient management. Fine-needle aspiration cytology (FNAC) is the gold-standard preoperative diagnostic tool, and The Bethesda System for Reporting Thyroid Cytopathology provides a standardized framework for interpretation. This 10-year retrospective study evaluated the malignancy risk in surgically treated patients with thyroid nodules classified as Bethesda Category III by comparing FNAC findings with histopathological outcomes.

Materials and methods

This observational retrospective study included patients with thyroid nodules classified as Bethesda Category III treated surgically at Shaukat Khanum Memorial Cancer Hospital and Research Centre (SKMCH & RC) Lahore between January 2014 and January 2024. Data were analyzed using SPSS version 20 (IBM Corp., Armonk, NY, US).

Results

A total of 44 patients were included in the study, with a mean age of 50.59 years. The majority were female 36 (81.8%) with 8 (18.2%) males. All patients underwent surgery, with 23 (52.3%) undergoing total thyroidectomy and 21 (47.7%) undergoing lobectomy. Final histopathological analysis revealed benign findings in 27 (61.4%) cases and malignancy in 17 (38.6%).

Conclusion

In our study, benign adenomatous nodules and papillary thyroid carcinoma had equal prevalence. The malignancy rate in Bethesda Category III nodules was 38.6%, higher than the previously reported range of 10% to 30%. This elevated risk may reflect the specialized cancer center setting in which the study was conducted, aligning with similar findings from other cancer centers. These results suggest that Bethesda Category III may carry a higher malignancy risk, warranting reconsideration of current management guidelines.

## Introduction

Thyroid cancer is the most common endocrine malignancy, and its global incidence has been steadily increasing over the past three decades [1]. The thyroid is a vital endocrine gland situated at the base of the neck, anterior to the trachea. Structurally, it consists of two wing-shaped lobes connected by a slender isthmus, which typically remains impalpable during physical examination. This gland plays a critical role in metabolic regulation, utilizing iodine to produce hormones that influence heart rate, blood pressure, body temperature, and basal metabolic rate. Its intricate functions underscore its importance in maintaining physiological balance and overall health [2].

Papillary thyroid carcinoma, the predominant subtype, accounts for approximately 84% of cases. Together with follicular (4%) and oncocytic (2%) variants, these subtypes constitute well-differentiated thyroid cancers, all of which arise from thyroid follicular cells. More aggressive forms of follicular cell-derived malignancies include poorly differentiated thyroid cancer (5%) and the rare but highly lethal anaplastic thyroid cancer (1%). In contrast, medullary thyroid cancer (4%) originates from parafollicular C cells [3].

The majority of well-differentiated thyroid cancers are asymptomatic and often detected incidentally during physical examinations or imaging studies. This highlights the critical importance of timely diagnosis and appropriate management of thyroid nodules to improve patient outcomes and reduce complications associated with advanced-stage disease. Early detection of thyroid malignancies enables effective intervention strategies, including surgical resection, radioactive iodine therapy, or hormonal treatment. With a spectrum of therapeutic options available, precise and accurate diagnosis remains pivotal in guiding optimal management pathways. Timely diagnosis and appropriate management of thyroid nodules are critically important to improve patient outcomes and mitigate complications associated with late-stage disease.

The past decade has ushered in a transformative era in thyroid cancer care, marked by significant advancements in diagnostic and management strategies. Internationally recognized ultrasound-based risk stratification systems for thyroid nodules have been introduced, aiming to minimize unnecessary biopsies. Additionally, less invasive approaches to managing low-risk thyroid cancers, including active surveillance and minimally invasive treatments, are gaining prominence as viable alternatives to traditional surgery [4]. Fine-needle aspiration cytology (FNAC) is widely recognized as the gold standard for the initial evaluation of thyroid nodules, particularly when paired with ultrasound guidance. Thyroid cytology proves to be a reliable, simple, and cost-effective first-line diagnostic procedure with high patient acceptance and with rare, usually easily treated, and not life-threatening complications [5]. Routine application of FNAC has decreased unnecessary referrals of thyroid nodules for surgical treatment [6].

Since its initial publication in 2010, the Bethesda System for Reporting Thyroid Cytopathology has revolutionized the evaluation of thyroid fine-needle aspiration (FNA) samples by providing a standardized, category-based framework [7]. This six-tiered reporting system has proven invaluable in reducing diagnostic inconsistencies and enhancing the correlation between cytological findings and histopathological outcomes, ultimately improving the accuracy and reliability of thyroid nodule assessments [8].

The Bethesda System for Reporting Thyroid Cytopathology (TBSRTC) 2023 emphasizes the use of six standardized diagnostic categories for reporting thyroid fine-needle aspiration (FNA) findings. The updated third edition addresses a prior source of confusion stemming from alternative terminologies for three diagnostic categories in earlier editions. To streamline reporting, TBSRTC 2023 has established a single, consistent designation for each category, discontinuing the use of terms such as “unsatisfactory,” “follicular lesion of undetermined significance,” and “suspicious for a follicular neoplasm.

The six diagnostic categories now recommended by TBSRTC 2023 are: (i) nondiagnostic, (ii) benign, (iii) atypia of undetermined significance (AUS), (iv) follicular neoplasm, (v) suspicious for malignancy (SFM), and (vi) malignant. The system continues to advocate for the use of these category names rather than numerical designations alone when reporting results or conducting scientific studies to avoid confusion with other numeral-based reporting systems such as the thyroid imaging and reporting system (TIRADS). While optional, appending a numerical designation alongside the category name (e.g., benign (Bethesda II), AUS (Bethesda III)) is considered an acceptable practice [9].

FNAC plays a pivotal role in distinguishing benign from malignant thyroid lesions. However, challenges arise when cytological findings fall into indeterminate categories, particularly Bethesda Category III. According to the TBSRTC, this category encompasses nodules with atypia or features of undetermined significance, making it difficult to classify them as clearly benign or malignant. The reported malignancy risk for Bethesda Category III nodules varies, ranging from 5-15% to as high as 12-30% in different studies [9-11].

 This ambiguity poses a dilemma for clinicians, as it leaves the malignancy risk uncertain, often necessitating further diagnostic procedures or more invasive interventions. Patients with Bethesda III nodules are typically managed with a range of options, including repeating FNAC, surgical intervention (either lobectomy or total thyroidectomy), or, in select cases, active surveillance. Each approach carries its risks and benefits, making it crucial to understand the underlying malignancy risk to guide decision-making [12,13]

The Bethesda consensus publication estimates that such a nodule would be associated with a low risk of malignancy (5-15%) and, in the absence of other suspicious features, could be managed with a repeat FNA. Conversely, Bethesda Category IV (follicular neoplasm or suspicious for follicular neoplasm) is thought to warrant surgery due to an estimated 15-30% risk of malignancy. In contrast to the Bethesda recommendations, the guidelines released by the American Association of Clinical Endocrinologists/Associazione Medici Endocrinology/European Thyroid Association (AACE/AME/ETA) Task Force combine Bethesda Categories III and IV (both defined as indeterminate). This group recommends surgery in most instances, with observation only in cases with favorable clinical, cytologic, and sonographic features [14].

The actual risk of malignancy is difficult to determine since a pathologic diagnosis is only available in the subset of patients selected for surgery. The risk of malignancy in Bethesda Category III (AUS/FLUS) is reported as high as 26.6-37.8% in a study from a dedicated cancer center [10].

Our 10-year retrospective study was designed to address this clinical uncertainty by investigating the malignancy risk associated with Bethesda III thyroid nodules in our center. By comparing FNAC findings with final histopathological outcomes, we aim to provide valuable insights into the likelihood of malignancy in these indeterminate cases. Additionally, this study seeks to offer evidence-based guidance for clinicians managing Bethesda III nodules, facilitating more informed decision-making regarding the need for surgery, repeat FNAC, or other interventions.

The findings from this study will not only contribute to the existing body of literature on thyroid cytopathology but also help refine local clinical guidelines and improve patient outcomes. By examining a decade of cases, we aim to offer a comprehensive evaluation of Bethesda III nodules, shedding light on the malignancy risk and the efficacy of various management strategies employed in our center. Ultimately, our goal is to assist clinicians in navigating the complexities of thyroid nodule management, ensuring that patients receive the most appropriate care.

## Materials and methods

Data design and source

This retrospective observational study was conducted at Shaukat Khanum Memorial Cancer Hospital and Research Centre (SKMCH & RC), Lahore, Pakistan, over 10 years (January 2014 to January 2024). The study was approved by the Institutional Review Board (Reference number: EX-12-03-24-01) and adhered to the Declaration of Helsinki. Medical records, ultrasound reports, FNAC findings, and histopathology reports were retrieved from the institutional database. Written informed consent was obtained for US-guided FNAC, which was performed as part of standard clinical care.

Study population

The study population comprised patients who underwent thyroid ultrasound, ultrasound-guided (US-guided FNAC), and subsequent thyroid surgery at Shaukat Khanum Memorial Cancer Hospital and Research Centre (SKMCH & RC). Inclusion criteria were patients aged 18 to 70 years with Bethesda Class III thyroid nodules identified on FNAC. Patients were excluded if they had missing preoperative FNAC or histopathological data, a history of multiple neck surgeries, or prior radiation exposure to the neck region.

Variables analyzed

The primary variables analyzed in this study included preoperative FNAC results, specifically focusing on nodules classified as Bethesda III under the Bethesda System for Reporting Thyroid Cytopathology. Postoperative histopathological outcomes were categorized as benign or malignant, with further sub-classifications for malignancy, such as papillary or follicular carcinoma, and benign conditions, including adenomatous or colloid nodules. Additionally, the surgical approach, whether lobectomy or total thyroidectomy, was examined to assess its relationship with the final histopathological findings.

Statistical analysis

Data were analyzed using SPSS version 20 (IBM Corp., Armonk, NY, US). The malignancy risk for Bethesda III nodules was calculated by comparing FNAC results with final histopathology findings. Categorical variables were expressed as frequencies and percentages while continuous variables were presented as means and standard deviations. The association between preoperative FNAC and histopathological outcomes was evaluated, with a p-value of <0.05 considered statistically significant. This analysis aimed to determine the malignancy risk of Bethesda III thyroid nodules and assess the appropriateness of surgical management strategies to optimize patient outcomes.

## Results

A total of 44 patients were included, with a mean age of 50.59 years. Notably, about half of the patients (21; 47.7%) belonged to the 41-60 age group, highlighting this range as the predominant age group for Bethesda III thyroid nodules in this cohort. The gender distribution showed a significant female predominance, with 36 (81.8%) participants being female and only 8 (18.2%) male (Table 1)

**Table 1:** Patient demographics

Demographic Characteristics	Frequency	Percentage
Males	8	18.2%
Females	36	81.8 %
Age Group 21-40	12	27.3 %
Age Group 41-60	21	47.7%
Age Group 61-80	11	25.0%

All patients underwent surgery: 23 (52.3%) underwent total thyroidectomy while 21 (47.7%) had lobectomy. Final histopathology revealed benign findings in 27 (61.4%) cases and malignancy in 17 (38.6%) (Table 2).

**Table 2:** Final histopathology FNAC: fine-needle aspiration cytology; AUS: atypia of undetermined significance; FLUS: follicular lesion of undetermined significance

FNAC	Final Histopathology After Surgery	
Bethesda III (AUS/FLUS)	Benign	Malignant
	27 (61.4 %)	17 (38.6%)

Within the malignant cases, 10 (22.7%) were identified as papillary thyroid carcinoma, making it the most common malignancy, followed by follicular thyroid carcinoma at 7 (15.9%). The benign category consisted of adenomatous nodules 10 (22.7%), benign follicular nodules 12 (27.3%), and colloid nodules 04 (9.1%). Interestingly, one patient (2.3% of the subset) exhibited normal thyroid tissue with no disease, emphasizing the variability of Bethesda III nodules. These findings contribute valuable insights into the malignancy risks and histopathological outcomes associated with Bethesda III thyroid nodules (Table 3).

**Table 3:** Histopathological outcomes

	Frequency (N)	Percentage (%)
Benign	27	61.4
Adenomatous nodules	10	22.7
Colloid nodules	4	9.1
Benign follicular nodules	12	27.3
Normal thyroid tissue	1	2.3
Malignant	17	38.6
Papillary thyroid carcinoma	10	22.7
Follicular thyroid carcinoma	7	15.9

## Discussion

This single-center retrospective study aimed to analyze the malignancy rates and characteristics of Bethesda Category III thyroid nodules, exploring their associations with clinical, histopathological, and laboratory variables in a regional population. The malignancy rates observed were 38.6% for category III nodules. Malignancy rates for Bethesda Category III and IV nodules vary widely across studies, with prior investigations reporting ranges of 15.7%-54.7% and 16.8%-72.4%, respectively [15,16].

The results of our study indicate a higher malignancy risk in Bethesda Category III thyroid nodules than the traditionally reported range, suggesting that indeterminate thyroid nodules may require a more nuanced approach. This finding aligns with similar studies from dedicated cancer centers, where higher malignancy rates for Bethesda III nodules are consistently reported [10].

This discrepancy from the general malignancy risk range could reflect the institutional focus on high-risk cases or differences in patient demographics, such as the notable female predominance (81.8%) seen in our cohort, aligning with global trends in thyroid cancer epidemiology.

In analyzing the malignancy rates within our Bethesda III cohort, papillary thyroid carcinoma emerged as the most prevalent malignant pathology (22.7%), followed by follicular thyroid carcinoma (15.9%). This distribution supports existing literature, which highlights papillary carcinoma as the most common thyroid malignancy [3]. However, our cohort's higher observed risk of malignancy suggests that Bethesda III nodules, even when classified as indeterminate, might carry a higher malignancy probability than currently assumed by general guidelines. This reinforces the necessity of thorough evaluation and potentially early surgical intervention for Bethesda III nodules, particularly in cancer center settings.

The clinical implications of these findings highlight the importance of tailoring management strategies based on institutional data and patient profiles. While current guidelines recommend a conservative approach with repeat FNAC for Bethesda III nodules, our study suggests that more aggressive management may be warranted in high-risk individuals. Repeat FNAC may provide limited diagnostic value in such settings, as the high rate of malignancy may not decrease with subsequent cytological testing. For high-risk patients, early surgical intervention, particularly lobectomy, may be more appropriate, given the notable rates of malignancy upon histopathological examination.

Our findings underscore the need for updated management guidelines that incorporate malignancy risk stratification for Bethesda III nodules in specialized cancer settings. By addressing the potential underestimation of malignancy risk, future guidelines can enhance diagnostic accuracy and reduce the likelihood of unnecessary interventions while optimizing outcomes for patients with thyroid nodules. Additionally, further research in diverse settings, especially multicenter studies, would contribute valuable data, supporting a more individualized, risk-adjusted approach to the management of Bethesda III thyroid nodules.

This study has several limitations that warrant consideration. First, as a retrospective chart review, it was inherently constrained by the inability to account for all clinical, histopathological, and radiological characteristics comprehensively. The absence of randomization and blinding introduces the potential for either exaggerating or underestimating observed associations.

Despite these challenges, efforts were made to minimize reporting bias by extracting data from the electronic hospital information system and cross-referencing records from all hospital visits. Another notable limitation was the lack of standardized ultrasonography reports for the study participants. As many scans were conducted at external healthcare facilities, retrieving raw imaging files was not feasible.

Additionally, the sample size of the study was relatively small. However, it remains consistent with similar investigations in the field. Finally, the study did not incorporate molecular testing for malignancy risk stratification, which could have provided additional insights. These limitations underscore the need for cautious interpretation of the findings and further research to validate the results.

## Conclusions

Our 10-year retrospective study of surgically treated Bethesda III thyroid nodules highlights a higher-than-expected malignancy risk, reflecting the variability seen in specialized cancer centers. The most common malignancies were papillary and follicular thyroid carcinoma while benign findings were predominantly adenomatous and follicular nodules. These findings challenge traditional management paradigms, emphasizing the need to reassess current guidelines to better reflect the elevated malignancy risk in high-risk populations. The study advocates for a more proactive surgical approach in selected cases and underscores the importance of multicenter research to enhance risk stratification and optimize treatment strategies for indeterminate thyroid nodules.
